# Case reports and the fight against cancer

**DOI:** 10.1186/1752-1947-2-39

**Published:** 2008-02-06

**Authors:** Elie G Dib, Michael R Kidd, Deborah C Saltman

**Affiliations:** 1 Sanford Cancer Center, University of South Dakota, 1020 West 18th Street, Sioux Falls, SD 57104, USA; 2 Discipline of General Practice, The University of Sydney, 37A Booth Street, Balmain, Sydney, NSW 2041, Australia; 3 Institute of Postgraduate Medicine, Brighton and Sussex Medical School, Mayfield House, University of Brighton, Falmer, Brighton BN1 9PH, UK

## Abstract

Some of the earliest case reports describing individual patients afflicted with cancer can be traced all the way back to the papyrus records of Ancient Egyptian medicine of approximately 1600 B.C.. Throughout the centuries physicians have continued the practice of writing case reports. Case reporting has provided significant advances in the knowledge of cancer on several fronts. It is without question that case reports do not replace well designed randomized clinical trials in advancing medical knowledge about cancerous diseases. However, case reports have their unique role in evidence-based medicine and often constitute the first line of evidence. This editorial reviews the many useful aspects of case reports and describes specific reports known to have revolutionized cancer management. *Journal of Medical Case Reports *is committed to publish well written case reports from around the world and be a source of inspiration for clinicians and scientists about newer research directions.

## Editorial

There has been a long descriptive history of case reporting in relation to cancer. In his book titled *Clinical Case Reporting in Evidence-Based Medicine*, Milos Jenicek eloquently describes case reports as the first line of evidence, where everything begins [1]. Some of the earliest case reports describing individual patients afflicted with cancer can be traced all the way back to the papyrus records of Ancient Egyptian medicine of approximately 1600 B.C.. These reports were the first recorded cases of incurable tumors of the breast [2]. Throughout the centuries physicians have continued the practice of writing case reports. Case reports of melanoma were described by Hippocrates in the fifth century B.C. and also by Rufus of Ephesus, a Greek physician, in the first century A.C. [3].

Another area where case reporting has provided significant advances in the knowledge of cancer has been in the identification of new types of cancer. For example, in January 1832, Thomas Hodgkin reported six cases to the Medical-Chirurgical Society of London, two of which were what we know today as Hodgkin's lymphoma [4]. In 1957, while in Uganda, Dennis P. Burkitt described a tumor that presented as a growth in the angle of the jaw of African children [5], later to be known as Burkitt's lymphoma. In 1960, Peter Nowell and David Hungerford published a report describing seven patients with chronic myeloid leukemia having the same "minute chromosome" later to be known Philadelphia chromosome [6]. In 1990, Farcet et al. described two patients with a new type of lymphoma, called Hepatosplenic T-Cell lymphoma [7], leading to more focused research of this new entity.

On the therapeutic front, the evolution of case reports to describe treatment entities is still evolving. To date the majority of reporting has been about adverse events. For example a case report about a patient with acute myeloid leukemia and nocardiosis revealed that high dose trimethoprim-sulfamethoxazole is a direct cause of significant myoclonus [8]. Another report revealed Aprepitant as a cause of Ifosfamide-induced encephalopathy [9]. More recently researchers have used the case report format to share unusual treatment combinations or responses. For example, Treon et al reported in 2004 about an interesting clinical response to sildenafil in Waldenström's macroglobulinemia [10].

It is without question that case reports do not replace well designed randomized clinical trials in testing new therapeutics. However, in cancer therapeutics the numbers of patients to conduct such studies may not be always recruitable. The advancement of cancer knowledge relies on a multitude of factors including molecular studies and preclinical models in order to study disease mechanism and potential targeted therapy: the kind of clinical information that could be gained through a series of case reports [11].

Another area where case reports about oncological matters have a role in the progress of medical science is in medical education [12]. Journal of Medical Case Reports (JMCR) is an open access journal that is committed to publishing high quality case reports from anywhere in the world and making them accessible to all. Through promoting the role of case reports in oncology, we hope to build a large prospective database of online case reports that will add to the aspects of oncology described in the evidence-based medical literature. This database will be a future resource allowing researchers to ask specific questions and study characteristics of uncommon events. JMCR will feed into an associated searchable database of case reports, and this database will serve as a clearing house of good case reports from all around the world. For example, by publishing every case report about spontaneous regression of metastatic renal cell carcinoma after debulking nephrectomy, a specific search of the database would help researchers in studying this particular and interesting phenomenon [13]. The prospective nature, of course would be in the data collection not retrieval. The planned aggregation of case reports has the potential to contribute to the ability to study newer risk factors associated with cancer. We recognize that case reports can serve as a substrate to further research many years after an original publication. For example, in 1886, Felix Fränkel described the first case report of pheochromocytoma in an 18 year-old woman with bilateral adrenal tumor. In 2007, almost 121 years later, Neumann et al. studied four living relatives of the same patient reported by Fränkel in 1886 and found that the patient had *RET *mutation and that her family had multiple endocrine neoplasia (MEN-2) [14].

In Medicine, questions in "research" (looking back) almost always start with patient encounters. Well written case reports will always be a source of inspiration for clinicians and scientists about newer research directions.

## Competing interests

ED is an Associate Editor and MK is the Editor-in-Chief of *Journal of Medical Case Reports*.
